# Surrounding Greenness and Exposure to Air Pollution During Pregnancy: An Analysis of Personal Monitoring Data

**DOI:** 10.1289/ehp.1104609

**Published:** 2012-05-30

**Authors:** Payam Dadvand, Audrey de Nazelle, Margarita Triguero-Mas, Anna Schembari, Marta Cirach, Elmira Amoly, Francesc Figueras, Xavier Basagaña, Bart Ostro, Mark Nieuwenhuijsen

**Affiliations:** 1 Centre for Research in Environmental Epidemiology (CREAL), Barcelona, Spain; 2 Municipal Institute of Medical Research (IMIM-Hospital del Mar), Barcelona, Spain; 3 CIBER Epidemiologia y Salud Pública (CIBERESP), Spain; 4 Department of Experimental and Health Sciences, Pompeu Fabra University, Barcelona, Spain; 5 Department of Maternal-Foetal Medicine, ICGON (Institut Clínic d´Obstetrícia, Ginecologia i Neonatologia), Hospital Clinic-IDIBAPS (Institut d´Investigacions Biomèdiques Agustí Pi i Sunyer), University of Barcelona, Barcelona, Spain; 6 Office of Environmental Health Hazard Assessment, California Environmental Protection Agency, Oakland, California, USA

**Keywords:** air pollution, greenness, green space, NDVI, personal exposure, personal monitoring, pregnancy, reproductive health, sustainability, urban green area

## Abstract

Background: Green spaces are reported to improve health status, including beneficial effects on pregnancy outcomes. Despite the suggestions of air pollution–related health benefits of green spaces, there is no available evidence on the impact of greenness on personal exposure to air pollution.

Objectives: We investigated the association between surrounding greenness and personal exposure to air pollution among pregnant women and to explore the potential mechanisms, if any, behind this association.

Methods: In total, 65 rounds of sampling were carried out for 54 pregnant women who resided in Barcelona during 2008–2009. Each round consisted of a 2-day measurement of particulate matter with aerodynamic diameter ≤ 2.5 μm (PM_2.5_) and a 1-week measurement of nitric oxides collected simultaneously at both the personal and microenvironmental levels. The study participants were also asked to fill out a time–microenvironment–activity diary during the sampling period. We used satellite retrievals to determine the surrounding greenness as the average of Normalized Difference Vegetation Index (NDVI) in a buffer of 100 m around each maternal residential address. We estimated the impact of surrounding greenness on personal exposure levels, home-outdoor and home-indoor pollutant levels, and maternal time-activity.

Results: Higher residential surrounding greenness was associated with lower personal, home-indoor, and home-outdoor PM_2.5_ levels, and more time spent at home-outdoor.

Conclusions: We found lower levels of personal exposure to air pollution among pregnant women residing in greener areas. This finding may be partly explained by lower home-indoor pollutant levels and more time spent in less polluted home-outdoor environment by pregnant women in greener areas.

About half of the global population now lives in cities and it is estimated that by 2030 three of every five persons will live in urban areas (Fuller and Gaston 2009; Smith and Guarnizo 2009). Urbanization has led more people to live in environments that are generally more polluted and less green (Cohen et al. 2005; Fuller and Gaston 2009; Grimm et al. 2008; Tzoulas et al. 2007). Urban air pollution is not only linked with increased mortality and morbidity in adults and children (Cohen et al. 2005) but also has been demonstrated to adversely affect fetal development (Shah et al. 2011; Vrijheid et al. 2011). For example, maternal exposure to ambient air pollution has been associated with the risk of low birth weight, preterm birth, intrauterine growth retardation, and congenital anomalies (Shah et al. 2011; Vrijheid et al. 2011). On the other hand, green spaces have been reported to improve both perceived and objective physical and mental health and well-being (Bowler et al. 2010; Maas 2008). More recently, studies have suggested that these spaces have some beneficial effects on pregnancy outcomes (Dadvand et al. 2011; Donovan et al. 2011). Specifically, these studies, conducted in the United States and Europe, reported that higher surrounding greenness of maternal residential address was positively associated with an increase in birth weight (Dadvand et al. 2011; Donovan et al. 2011).

Although, the underlying pathways of the effects of green spaces on health are not fully understood, increased physical activity, increased social contacts, reduced psychophysiological stress and depression, decreased noise, microclimate regulation (i.e. moderation of ambient temperature and urban heat island effects), and reduced air pollution levels have been suggested to be involved (Bowler et al. 2010; Gill et al. 2007; Greenspace Scotland 2008; Health Council of the Netherlands 2004; Lee and Maheswaran 2010; Maas 2008; Maas et al. 2009a, 2009b; Nowak et al. 2006; Whitford et al. 2001).

Green spaces reduce air pollution by direct and indirect mechanisms (Givoni 1991). The direct mechanism is via the filtering effect of plants, principally based on dry deposition of pollutants (both particles and gases) through stomata uptake or nonstomata deposition on plant surfaces (Akbari 2002; Givoni 1991; Nowak et al. 2006; Paoletti et al. 2011). The indirect effect is mediated by improving urban ventilation which in turn increases the dispersal of the pollutants (Givoni 1991) and also by decreasing ambient temperature, which in turn decelerates the smog formation (Akbari 2002).

Although the ability of green spaces in improving air quality has been widely reported and is suggested to be one of the mechanisms behind the health benefits of the green spaces (Akbari 2002; Givoni 1991; Maas 2008; Nowak et al. 2006; Paoletti et al. 2011; Su et al. 2009, 2011), the available evidence on the impact of surrounding greenness on personal exposure to air pollution is nonexistent. This impact, if any, is of importance because personal exposure to air pollution is a complex function of microenvironmental pollutant levels and personal behavior, both of which could be associated with surrounding greenness.

The aim of our study was to investigate the association between surrounding greenness and personal exposure to air pollution among pregnant women and to explore the potential mechanisms behind this association, if any.

## Materials and Methods

*Study population.* The study population consisted of 54 Spanish-speaking pregnant women who lived in Barcelona, Spain, from November 2008 to November 2009. The women were recruited from those attending the obstetrics department of the Hospital Clinic of Barcelona for their first, second, or third pregnancy visits. Hospital Clinic of Barcelona is a major university hospital in Barcelona with a catchment area of about one million inhabitants (Figueras et al. 2008). We randomly selected 16 dates from 2008 to 2009, and pregnant women who had an appointment with the hospital within those dates were contacted by phone and invited to participate in the study (*n* = 434). Women who were interested in participating in the study were then recruited by the study technician on their next visit to the hospital.

*Air pollution sampling.* Nitric oxides (NO_x_) and particulate matter with aerodynamic diameter ≤ 2.5 μm (PM_2.5_) were used as indicators of air pollution in our study. Each sampling round consisted of a 2-day measurement of PM_2.5_ and a 1-week measurement of NO_x_ simultaneously for both personal and microenvironmental levels. For 11 participants, we carried out two sampling rounds.

Personal exposure levels. During the first 48-hr of the 1-week sampling period, which always occurred on weekdays, participants were asked to wear a small backpack containing a portable particle monitor when they were awake and to place the monitor next to their bed when they were sleeping. The portable particle pump (BGI400S pump; BGI Incorporated, Waltham, MA, USA) operated continuously during this period, collecting PM_2.5_ using a GK2.05 sampler (BGI Incorporated) and a 37-mm Teflon filter (Pall Corporation, East Hills, NY, USA) with a 50% cut-point of 2.5 μm at a flow rate of 4 L/min (van Roosbroeck et al. 2006). The flow rate was adjusted at the beginning of each sampling round to 4 L/min with a rotameter (model RM67, BGI Incorporated) and checked at the end of that sampling round to make sure that it had remained at 4 L/min during the course of sampling. In addition, the participants were asked to wear a passive sampler (PS-100; Ogawa & Co. USA, Inc., Pompano Beach, FL, USA) during the entire week to measure personal levels of NO_x_.

Microenvironmental levels. Pollutant levels were measured within (home-indoor levels) and outside (home-outdoor levels) the participants’ homes. The home-indoor monitors were placed in the living room, while the home-outdoor monitors were installed outside a window or in the terrace/balcony of maternal homes. Similar to the personal measurements, the microenvironmental PM_2.5_ concentrations were measured by a portable particle monitor for the first two days and NO_x_ levels were measured by a passive sampler during the whole week of the sampling round at fixed locations.

*Time–activity data.* To characterize the time that the participants spent in various microenvironments and activities, we used a modified version of the EXPOLIS (Air Pollution Exposure Distributions of Adult Urban Populations in Europe) Time–Microenvironment–Activity Diary (TMAD) (Hänninen et al. 2004), which we enhanced to include self-reported levels of physical activity for each activity. Each participant was asked to record the microenvironment–activity category for every 30 min of her sampling period. The microenvironment categories included in the TMAD were walk, bike, motorcycle, car/taxi, bus, tram, metro, and train (“in transfer” categories) and indoors at home, outdoors at home, indoors at work, outdoors at work, indoors at another location, and outdoors at another location (“not in transfer” categories) (Hänninen et al. 2004). The diary also included data on smoking (active or passive), use of a nonelectrical heater, and the application of the kitchen hood while cooking with nonelectrical appliances [see Supplemental Material, Figure S1 (http://dx.doi.org/10.1289/ehp.1104609)].

*Surrounding greenness.* To determine the surrounding greenness, we used the Normalized Difference Vegetation Index (NDVI) derived from the Landsat Enhanced Thematic Mapper Plus (ETM+) data at 30 m × 30 m resolution [see Supplemental Material, Figure S2 (http://dx.doi.org/10.1289/ehp.1104609)] (Dadvand et al. 2011). The ETM+ Landsat data were acquired for 10 August 2000 covering path 197 and row 031 (i.e., the scope of Barcelona). NDVI is an indicator of greenness based on land surface reflectance of visible (red) and near-infrared parts of spectrum (Weier and Herring 2011). NDVI ranges between –1 and 1 with higher numbers indicating more greenness. Surrounding greenness was abstracted as the average of NDVI in a buffer of 100 m around each maternal place of residence geocoded according to the address at the sampling time. The choice of buffer size was based on the findings of two recent studies on the association between green spaces and pregnancy outcomes showing that only immediate surrounding greenness (buffers of 50 m and 100 m) was related with pregnancy outcomes (Dadvand et al. 2011; Donovan et al. 2011). This choice was also informed by previous reports suggesting that the impact of green spaces on reducing air pollution levels mostly occurs within immediate vicinity of these spaces and decays rapidly as the distance from green spaces increases (Givoni 1991).

*Statistical analysis.* We estimated the impact of surrounding greenness on personal exposure levels, home-outdoor and home-indoor pollutant levels, and maternal time-activity. For these associations, we developed linear regression models to estimate the change in the outcome associated with an interquartile range (IQR) increase in surrounding greenness (NDVI). We used the measured values for the outcomes without transformation to produce interpretable results and capture the entire range of the variation. We checked the homoscedasticity and normality of the regression residuals and confirmed the robustness of our regressions to these assumptions (data not shown).

Personal exposure levels. For each pollutant, we first modeled personal exposure levels against surrounding greenness using the regionwide average of pollutant levels during the sampling period for each participant as an offset to account for temporal variation in background pollutant levels. To calculate regionwide averages, the daily pollutant levels, which were measured by seven monitoring stations for NO_x_ and three monitoring stations for PM_2.5_ across Barcelona, were averaged for each day of the study period. Each participant was then assigned the mean of the daily pollutant levels for her sampling period.

We further adjusted the estimates for personal exposure for the time spent at home (sum of time spent at home-indoor and home-outdoor), time spent in transfer, smoking (active and passive), use of gas-cooking appliances, and the MEDEA [Construcción de un índice de privación a partir de datos censales en grandes ciudades españolas (Proyecto MEDEA)] index of neighborhood deprivation (Domínguez-Berjón et al. 2008). Maternal socioeconomic status (SES) could be associated with both air pollution levels (personal, indoor, and outdoor) and surrounding greenness. We included indicators of SES at both individual and neighborhood levels in our analyses. For individual SES we adjusted for number of inhabitants at home (an indicator for overcrowding), as well as use of gas-cooking appliances and maternal exposure to tobacco smoke, which might also be indicators of SES. For the neighborhood SES, we used the MEDEA index which measures deprivation at the census tract level based on five domains including percentage of manual workers, temporary workers, people with low education (overall), young population with low education, and unemployment (Domínguez-Berjón et al. 2008). These domains have been shown to explain 75% of the variability of all socioeconomic variables available in the Spanish census (Domínguez-Berjón et al. 2008). In the 2001 Census, there were 1,491 census tracts across the city of Barcelona with a median area of 0.02 km^2^ and population of 992.

Microenvironmental pollutant levels. Pollutant-specific models were developed using home-outdoor pollutant levels as the outcome; surrounding greenness, surrounding traffic intensity, the height of the monitor, and the MEDEA index of neighborhood deprivation as predictors; and the regionwide average of pollutant levels during the sampling period for each participant as an offset. Traffic flow in each street was obtained from traffic flow models developed by the Barcelona City Council (Ajuntament de Barcelona 2007). For each participant, the lengths of all streets falling within the buffer of 100 m around her place of residence were abstracted and multiplied by the corresponding flow for each street. The surrounding traffic intensity (vehicle kilometers traveled) was then calculated by summing these values. The Pearson’s correlation coefficient between surrounding greenness and surrounding traffic intensity within the 100 m buffer was –0.08 (*p* = 0.51).

For each pollutant, we modeled home-indoor levels against the surrounding greenness, smoking, use of gas-cooking appliances, the number of inhabitants at home, the temperature at home-indoor on the first day of sampling round, and the MEDEA index of neighborhood deprivation, while using regionwide average of pollutant levels during the sampling period for each participant as an offset.

Time–activity pattern. To estimate the impact of surrounding greenness on maternal time-activity, we developed models with home-outdoor time as the outcome and surrounding greenness, season of sampling, and the MEDEA index of neighborhood deprivation as predictors. The same modeling framework was repeated using home-indoor time as the outcome.

*Sensitivity analyses.* Buffer size for abstracting surrounding greenness. We chose a buffer of 100 m around each maternal residence to calculate surrounding greenness. To evaluate the robustness of our findings to alterations in this buffer size, we abstracted the surrounding greenness in buffers of 250 m and 500 m and repeated all the aforementioned analyses using these alternative exposure variables plus alternative traffic intensity variables for buffers of 250 m and 500 m, respectively. The results were expressed for a change in the IQR of the surrounding greenness of the subjects in each buffer size.

Surrounding greenness of the working place. Of the 54 study participants, 18 were working outside of their homes during their sampling period. The working address was known and geocoded for 17 participants and the surrounding greenness (100-m buffer) was extracted for these addresses. We calculated a home–work surrounding greenness index by averaging the surrounding greenness of maternal residential and working addresses weighted by the time that participants spent in each place. We then modeled personal exposure levels against this index together with smoking, use of gas-cooking appliances, time spent in transfer, MEDEA index of neighborhood deprivation, and a binary (yes/no) variable determining whether the participant was working during the sampling period.

Repeated sampling rounds. Our analysis was based on 65 observations from 54 participants including two sampling rounds for a subset of 11 participants. We considered a gap of at least 1 month between two sampling rounds for each participant to minimize the effect of repetitive measurements. To evaluate the effect of second sampling rounds on our findings, we carried out a sensitivity analysis including only the first sampling round for each participant in the models for personal, home-indoor, and home-outdoor levels.

*Ethics approval.* Ethics approval (No. 2008/3115/I) was obtained from the Clinical Research Ethical Committee of the Parc de Salut MAR, Barcelona, Spain, to carry out this study. All participants gave their written informed consent prior to the study.

## Results

Of the 434 women contacted by the study technicians, 54 agreed to participate in the study. A total of 65 air pollution measurements were carried out, with two sampling rounds for 11 participants and a single round for 43 participants. One of the participants (one sample) with metallurgy processing nearby her home was identified as an outlier and excluded from the analyses. One sampling round was performed during the first trimester, 23 during the second trimester, and 41 during the third trimester of participants’ pregnancies. As presented in Table 1, 24 (44.4%) participants reported exposure to tobacco smoke (passive or active) and 46 (85.2%) participants reported use of gas-cooking appliances during the sampling period.

**Table 1:** Descriptive statistics of the study participants (*n* = 54).

Variable	Median (IQR)^a^
Personal exposure (μg/m3)	
PM2.5b	23.2 (9.9)
NOxc	56.6 (34.1)
Home-indoor levels (μg/m3)	
PM2.5b	20.1 (13.5)
NOxc	55.1 (40.8)
Home-outdoor levels (μg/m3)	
PM2.5b	17.7 (9.5)
NOxc	49.9 (26.3)
Home surrounding NDVI average	
100 m buffer	–0.283 (0.049)
250 m buffer	–0.275 (0.031)
500 m buffer	–0.267 (0.042)
Time spent (min/day)	
Home-indoor	960.0 (264.6)
Home-outdoor	1.9 (15)
Transfer	67.5 (50.8)
Cooking fuel [n (%)]	
Natural gas	39 (72.2)
Butane gas	7 (13.0)
Electric/not gas	8 (14.8)
Smoking [n (%)]	
Yes	24 (44.4)
No	30 (55.6)
Home-indoor temperature (ºC)d	24.5 (5.0)
Monitor height (m)	9 (6)
MEDEA index	0.20 (1.44)
No. of inhabitants at home	3 (2)
aData are median (IQR), unless othewise noted. bCumulative concentration for 2 days of sampling. cCumulative concentration for 1 week of sampling. dMeasured on the first day of sampling.	

Median home-indoor pollutant levels were generally higher than median home-outdoor levels (Wilcoxon signed-rank test *p*-value of 0.06 for PM_2.5_ and < 0.01 for NO_x_) (Table 1). On average, participants spent 73.3% of their time at home-indoors and 1.1% at home-outdoors. Participants working outside of the home spent 14.4% of their time at work (including indoor and outdoor time). The median (minimum and maximum) of surrounding NDVI (100 m buffer) was –0.28 (–0.32, –0.14) for residential addresses and –0.27 (–0.36, –0.21) for places of work.

Higher residential surrounding greenness was associated with lower average levels of personal PM_2.5_ exposures in both unadjusted and adjusted models (Table 2). The increase in surrounding greenness of maternal residential addresses was also associated with a decrease in the average home-indoor and home-outdoor PM_2.5_ levels, with a statistically significant (*p* < 0.05) association for home-indoor PM_2.5_ and nearly statistically significant association for home-outdoor PM_2.5_ (Table 2). For NO_x_, none of the associations attained statistical significance, but their directions were consistent with those of PM_2.5_ (Table 2).

**Table 2:** Regression coefficients (95% CIs) of change in personal exposure and microenvironmental pollutant levels (μg/m^3^) associated with an IQR^a^ increase in the average NDVI within the buffers of 100 m, 250 m, and 500 m around maternal residential addresses.

Surrounding greenness										
100-m buffer			250-m buffer			500-m buffer		
Measurements	Regression coefficient (95% CI)	p-Value	Regression coefficient (95% CI)	p-Value	Regression coefficient (95% CI)	p-Value
Personal (unadjusted)												
PM2.5		–5.2 (–9.4, –0.9)		0.02		–2.4 (–5.0, 0.1)		0.06		–2.8 (–5.8, 0.3)		0.08
NOx		–2.6 (–15.3, 10.1)		0.68		–2.3 (–9.7, 5.1)		0.54		–3.2 (–12.4, 6.0)		0.49
Personal (adjusted)b												
PM2.5		–5.9 (–10.0, –1.8)		< 0.01		–2.4 (–4.8, 0.0)		0.05		–2.3 (–5.1, 0.5)		0.11
NOx		–5.1 (–18.6, 8.4)		0.45		–3.0 (–10.7, 4.6)		0.43		–3.6 (–12.9, 5.7)		0.44
Home-indoorc												
PM2.5		–6.1 (–10.6, –1.6)		< 0.01		–1.9 (–4.6, 0.8)		0.17		–2.3 (–5.5, 0.9)		0.15
NOx		–9.5 (–24.4, 5.3)		0.20		–4.5 (–13.3, 4.2)		0.31		–6.7 (–17.3, 3.9)		0.21
Home-outdoord												
PM2.5		–4.4 (–9.5, 0.7)		0.08		–3.2 (–6.6, 0.2)		0.07		–5.5 (–10.5, –0.4)		0.04
NOx		–5.8 (–17.6, 6.0)		0.33		–5.3 (–14.0, 3.4)		0.23		–5.6 (–19.5, 8.3)		0.43
a0.049 for 100 m buffer, 0.031 for 250 m buffer, and 0.042 for 500 m buffer. bAdjusted for the time spent at home (sum of time spent at home-indoor and home-outdoor), smoking (active and passive), use of gas-cooking appliances, time spent in transfer, and MEDEA index of neighborhood deprivation. cAdjusted for the temperature at home-indoors on the first day of sampling round, the use of gas-cooking appliances, smoking (active and passive), the number of inhabitants, and MEDEA index of neighborhood deprivation. dAdjusted for the traffic intensity in the buffer of 100 m around maternal residential address, the height of the monitor, and MEDEA index of neighborhood deprivation.												

For maternal time–activity patterns, an interquartile increase in surrounding greenness was associated with a nearly statistically significant 12-min/day increase in time spent home-outdoors [95% confidence interval (CI): 0, 24; *p* = 0.07] but was not significantly associated with time spent home-indoors (6-min increase, 95% CI: –66, 71; *p* = 0.94). Trimester of sampling was not significantly associated with the time spent at home-indoors or home-outdoors (Kruskal–Wallis test *p*-values of 0.44 and 0.24, respectively).

The results from the sensitivity analyses evaluating the impact of different buffer sizes on our findings were generally consistent with our main findings; however, for home-outdoor PM_2.5_, the association became stronger and attained statistical significance when surrounding greenness was based on a 500-m buffer (Table 2). For personal PM_2.5_ exposure, the association seemed to weaken with larger buffer sizes, with a statistically nonsignificant association for a 500-m buffer. For NO_x_ associations remained statistically nonsignificant for all buffer sizes (Table 2).

An IQR (0.57) increase in the home–work surrounding greenness index was associated with a 5.4-μg/m^3^ decrease (95% CI: –10.2, –0.6) in average personal PM_2.5_ levels and a statistically nonsignificant 5.3-μg/m^3^ decrease (95% CI: –17.8, 7.3) in average personal NO_x_ levels.

The exclusion of data from second sampling rounds for the 11 participants with two sampling rounds did not result in a notable change in our findings [see Supplemental Material, Table S1 (http://dx.doi.org/10.1289/ehp.1104609)].

## Discussion

To our knowledge, this is the first study to report on the association between surrounding greenness and measured personal exposure to air pollution. Our study sample consisted of 54 pregnant women who resided in Barcelona from 2008 to 2009. The analysis was based on data obtained from time–activity diaries and air pollution sampling rounds that included personal and microenvironmental measurements of PM_2.5_ (2 days) and NO_x_ (1 week). To measure surrounding greenness, we used averages of NDVI within a buffer of 100 m around each maternal residential address. Higher residential surrounding greenness was associated with lower personal home-indoor and home-outdoor PM_2.5_ levels and with more time spent home-outdoor. The results for NO_x_ were less conclusive.

We observed a reduction in average personal exposure to PM_2.5_ associated with an increase in greenness surrounding the maternal residential addresses. There was also an indication for a similar association between surrounding greenness and average personal exposure to NO_x_, but the association did not attain statistical significance. NO_x_ participate in complex photochemical reactions with volatile organic compounds (VOCs) emitted by plants (Kesselmeier and Staudt 1999) and this could make identifying trends for NO_x_ and green space more challenging.

We are unaware of previous studies on the impact of surrounding greenness on measured personal exposure among pregnant women or in the general population. In a recent analysis of modeled ambient concentrations of pollutants [nitrogen dioxide (NO_2_)], PM_2.5_, and ozone] in and around parks in the Los Angeles metropolitan area, California, Su et al. (2011) found that PM_2.5_ and NO_2_ were higher in neighborhoods adjacent to the parks. Their finding was likely due to the very particular circumstances of the Los Angeles area where parks are predominantly located near highways with heavy traffic. Our measure is different in that the presence of green space as indicated by the NDVI index is a more continuous measure evenly distributed across the city and is not related to proximity to roadways with heavy traffic. In addition, our multivariate analysis of home-outdoor concentrations accounted for traffic intensity in the immediate surroundings of the homes (100-m buffer). In the Los Angeles study, Su et al. (2011) did find, however, that modeled pollutants were lower inside the parks compared with the region as a whole, which perhaps better reflects the greenery in the immediate surroundings of the residences of our participants as measured by the NDVI.

The findings for home-outdoor PM_2.5_ levels are in line with the available evidence that has shown that green spaces reduce air pollutant levels by filtering air pollutants, improving urban circulation, and reducing ambient temperature (Akbari 2002; Givoni 1991; Nowak et al. 2006; Paoletti et al. 2011; Su et al. 2011). To our knowledge, there is no previous report quantifying the association between surrounding greenness and home-indoor pollutant levels. Our observed reduction in home-indoor PM_2.5_ levels could have been secondary to decreases in home-outdoor PM_2.5_ levels.

The study participants spent much of their time home-indoors (73.3%) and our observed lower home-indoor pollutant levels for participants with higher degrees of surrounding greenness can partly explain their lower personal exposure levels. The impact of lower levels of indoor pollution in greener areas is supported by our observed strong correlation between personal and home-indoor PM_2.5_ levels (Pearson’s correlation coefficient of 0.78, *p* < 0.01). Furthermore, higher degrees of surrounding greenness were associated with a nearly significant 86% increase in the time that our participants on average spent home-outdoors (14 min). This increase was in line with previous reports suggesting an increase in outdoor physical activity in relation with higher degrees of surrounding greenness (Ellaway et al. 2005). Because home-outdoor pollutant levels were lower than those of home-indoor levels, this increase in time spent in less polluted home-outdoor environment could result in lower personal exposure levels.

We conducted a range of sensitivity analyses, including testing the effect of the buffer size for calculating surrounding greenness on our investigated associations. Our findings appeared to be robust to buffer size; however, for personal PM_2.5_ exposure the associations seemed to weaken with larger buffer sizes. This pattern is in line with findings of previous reports showing that only immediate surrounding greenness was associated with pregnancy outcomes (Dadvand et al. 2011; Donovan et al. 2011). We also conducted a separate analysis of associations with surrounding greenness at work as well as at home using a weighted average of NDVI at both locations for the subset of participants who worked outside of the home, and the results were consistent with our main findings.

Our study faced some limitations. Indoor greenness (e.g., houseplants) has been suggested to improve indoor air quality (Claudio 2011). We did not have data on indoor greenness and were not able to address it in our analyses. This might confound or modify our estimated associations. Furthermore, our study used remote-sensing–derived NDVI to measure surrounding greenness. Application of this objective measure of greenness enabled our study to take account of small-scale green spaces (e.g., home gardens, street trees, and green verges) in a standardized way; however, NDVI does not distinguish between different types of vegetation. This distinction may be important because there is some evidence that the effect of green spaces on air pollutants is vegetation-dependent, with trees being the most effective and grasses being the least effective (Givoni 1991). The inability of NDVI to distinguish between different vegetation types may have been a source of exposure misclassification in our study. The NDVI map used in this study was based on remote sensing data collected in 2000 while our study was carried out during 2008–2009. To evaluate the temporal validity of our NDVI map, we applied an ecologic map of Barcelona for the year 2004 (Centre for Ecological Research and Forestry Application 2004) to abstract the percentage of the green areas over grids of 100 m × 100 m across the Barcelona. We also abstracted the averages of NDVI over the same grids. The strong correlation (Pearson’s correlation coefficient of 0.81, *p* < 0.001) between these two measures could suggest a satisfactory temporal validity of NDVI map to estimate the surrounding greenness for our study.

Our classification of the time spent by the participants in each microenvironment was based on the TMAD records. Although the participants were asked to provide a TMAD record every 30 min, which could minimize the problem of recall, there might still be some misclassifications if the women filled out TMAD after longer periods of time (e.g., if they left their TMAD at home when they went to work). We did not equip our participants with global positioning systems (GPSs), so we were not able to validate TMAD records on time spent in microenvironments. This would be helpful in future studies.

## Conclusions

We investigated the association between surrounding greenness of the place of residence and personal exposure to PM_2.5_ and NO_x_ among a sample of 54 pregnant women in Barcelona (2008–2009) and observed lower average PM_2.5_ exposure levels associated with higher surrounding greenness. This finding could be partly explained by lower home-indoor pollutant levels (where participants spent most of their time) and more time spent in less polluted home-outdoor environments by the participants in greener areas.

The time–activity patterns of our study sample are not necessarily generalizable to the general population, especially since most of the samplings were carried out during the third trimester of pregnancy when the mothers would be less mobile in comparison with the general population. Because the time–activity pattern plays an important role in personal exposure to air pollutants, the extrapolation of our findings to the general population should be considered with caution. Nonetheless, our findings are suggestive for a possible beneficial effect of green spaces in reducing exposure to air pollution among the general population (in particular for less mobile groups such as the elderly and children) and would therefore encourage further investigation of this effect. This impact would also be of interest for policy makers in planning sustainable development of urban environments. We recommend future studies to be based on larger samples from general population and rely on better characterization of surrounding greenness (i.e., vegetation type) and take account of indoor greenness.

## Supplemental Material

(176 KB) PDF
