# Supplementary material for: Surrounding Greenness and Exposure to Air Pollution During Pregnancy: An Analysis of Personal Monitoring Data
Source: Environ Health Perspect. 2012 May 30;120(9):1286–90. doi: 10.1289/ehp.1104609 (PMC3440116; doi:10.1289/ehp.1104609)
Supplement: (176 KB) PDF [file ehp.1104609.s001.pdf]

Supplemental Materials

Surrounding Greenness and Exposure to Air Pollution During Pregnancy: An Analysis of Personal Monitoring Data

Payam Dadvand [1,2,3]; Audrey de Nazelle [1,2,3]; Margarita Triguero-Mas [1]; Anna Schembari [1,2,3,4]; Marta Cirach [1]; Elmira Amoly [4]; Francesc Figueras [5]; Xavier Basagaña [1,2,3]; Bart Ostro[1,6]; Mark Nieuwenhuijsen [1,2,3]

[1] Centre for Research in Environmental Epidemiology (CREAL), Barcelona, Spain.

[2] Municipal Institute of Medical Research (IMIM-Hospital del Mar), Barcelona, Spain.

[3] CIBER Epidemiologia y Salud Pública (CIBERESP), Spain.

[4] Department of Experimental and Health Sciences, Pompeu Fabra University, Spain.

[5] Department of Maternal-Foetal Medicine, ICGON, Hospital Clinic-IDIBAPS, University of Barcelona, Spain.

[6] Office of Environmental Health Hazard Assessment, California Environmental Protection Agency, Oakland, CA, USA.

Table of contents

Supplemental Material, Table S1 Page 3

Supplemental Material, Figure S1 Page 4

Supplemental Material, Figure S2 Page 5

Supplemental Material, Table S1. Regression coefficients (95% confidence interval (CI)) of change in personal and microenvironmental pollutant levels (μg/m^3) due to an inter-quartile range increase in the average NDVI within a buffer of 100m around maternal residential addresses separately for 64 sampling rounds (including two sampling rounds for 11 participants) and 53 sampling rounds (one sampling round per participant).

**Measurements**	**$PM_{2.5}$**		**NO_x**	
	**64 sampling rounds**	**53 sampling rounds**	**64 sampling rounds**	**53 sampling rounds**
**Personal (Un-adjusted)**	-5.2 (-9.4, -0.9)	-6.0 (-10.7, -1.3)	-2.6 (-15.3, 10.1)	-3.1 (-16.5, 10.2)
**Personal (Adjusted)**[a]	-5.9 (-10.0, -1.8)	-6.1 (-10.7, -1.4)	-5.1 (-18.6, 8.4)	-3.7 (-18.4, 10.9)
**Home-indoor**[b]	-6.1 (-10.6, -1.6)	-6.2 (-11.1, -1.3)	-9.5 (-24.4, 5.3)	-7.9 (-24.8, 8.9)
**Home-outdoor**[c]	-4.4 (-9.5, 0.7)	-5.5 (-11.2, 0.2)	-5.8 (-17.6, 6.0)	-1.9 (-11.8, 8.0)

a Adjusted for time spent at home (sum of time spent at home-indoor and home-outdoor), smoking (active and passive), use of gas cooking appliances, time spent in transfer, and MEDEA index of neighborhood deprivation.

b Adjusted for the temperature at home-indoor on the first day of sampling round, the use of gas cooking appliances, smoking (active and passive), the number of inhabitants, and MEDEA index of neighborhood deprivation.

c Adjusted for the traffic intensity in the buffer of 100m around maternal residential address, the height of the monitor, and MEDEA index of neighborhood deprivation.

Day:

Fill in all the cells divided in 30 minutes fractions

HOUR		PLACE (NO TRANSPORT)						TRANSPORT									ACTIVITIES					PHYSICAL ACTIVITY LEVEL			
		I am at...						Travelling by...									I am...								
		HOME		WORK		OTHERS		walk	bike	motorcycle	car/ taxi	bus	tram	underground	train	others	Smoking	cooking or in a room where somebody is cooking		in a room where ...		INACTIVE	LOW	MODERATE	HIGH
																		and the hood is...		a (non-electric) heater is on	Somebody is smoking				
		indoors	outdoors	indoors	outdoors	indoors	outdoors											on	off						
	00																								
06	30																								
	00																								
07	30																								
	00																								
08	30																								
	00																								
09	30																								
	00																								
10	30																								
	00																								
11	30																								
	00																								
12	30																								
	00																								
13	30																								
	00																								
14	30																								
	00																								
15	30																								
	00																								
16	30																								
	00																								
17	30																								
	00																								
18	30																								
	00																								
19	30																								
	00																								
20	30																								
	00																								
21	30																								
	00																								
22	30																								
	00																								
23	30																								
	00																								
00	30																								
	00																								
01	30																								
	00																								
02	30																								
	00																								
03	30																								
	00																								
04	30																								
	00																								
05	30																								

Supplemental Material, Figure S1. Time-Microenvironment-Activity-Diary.

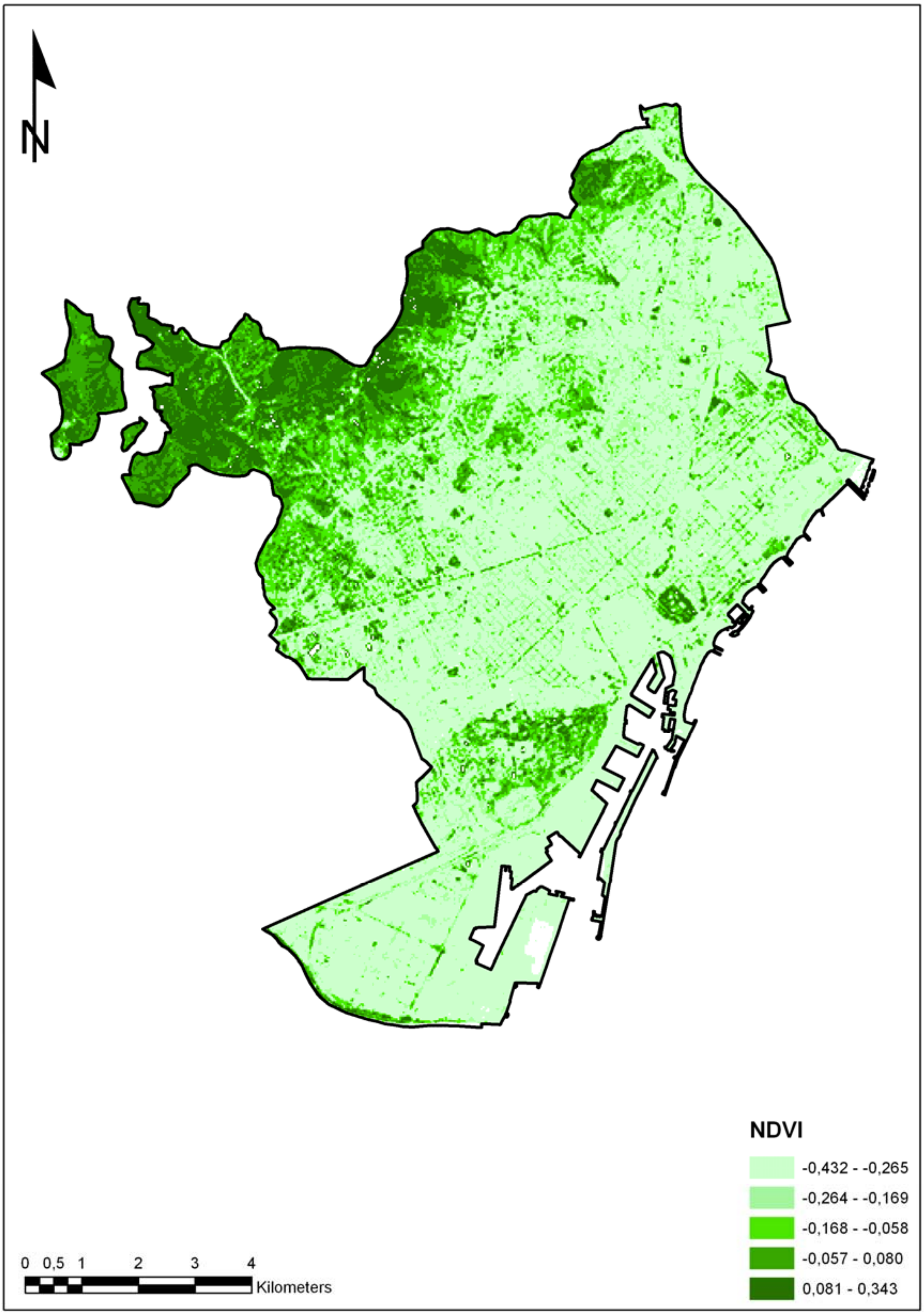

Supplemental Material, Figure S2. Map of Normalized Difference Vegetation Index across Barcelona, August 10th, 2000.
